# CIRCULATING VIRAL RESPIRATORY PATHOGENS AS CAUSATIVE AGENT FOR SEVERE ACUTE RESPIRATORY INFECTIONS IN MOROCCO: A SYSTEMATIC REVIEW

**DOI:** 10.21010/Ajidv19i2S.12

**Published:** 2025-10-17

**Authors:** KHARBACH Ahmed, BABA Mohamed Amine, OUBAASRI Ahmed, ABDA Naima, KHALIS Mohamed, OUAALAYA El Hassane, BIGI Soufiane, WAKRIM Soukaina, IDRISSI Karim Sbai, BELYAMANI Lahcen, RAZINE Rachid, TRIKI Soumia, MALIK Mamunur Rahman, OBTEL Majdouline

**Affiliations:** *1 Laboratory of Biostatistics, Clinical Research and Epidemiology, Faculty of Medicine and Pharmacy of Rabat, Mohamed V University, Rabat 10100, Morocco; 2** High Institute of Nursing Professions and Technical Health, Agadir, Morocco; 3 Laboratory of Cell Biology and Molecular Genetics, Department of Biology, Faculty of Sciences, Ibn Zohr University, Agadir, Morocco; 4 Ibn Zohr University, Faculty of Medicine and Pharmacy, REGNE Research Laboratory, Agadir, Morocco; 5 High Institute of Nursing Professions and Technical Health, Guelmim, Morocco; 6 Ibn Zohr University, Faculty of Medicine and Pharmacy, Agadir, Morocco; 7 Department of Epidemiology and Public Health of the Faculty of Medicine and Pharmacy, University Mohammed I of Oujda, Morocco; 8 High Institute of Nursing Professions and Technical Health, Rabat, Morocco; 9 University Mohammed VI of Health and Sciences in Casablanca, Morocco; 10 Center of Research and Innovation Mohammed VI in Rabat, Morocco; 11 Department of Public Health, Faculty of Medicine and Pharmacy, Mohammed V University in Rabat, Morocco; 12 WHO Country Office, Rabat, Morocco; 13 Eastern Mediterranean Regional Office, World Health Organisation, Cairo, Egypt; 14 Physiology and Physiopathology Research Team, Research Centre of Human Pathologies, Genomics, Faculty of Sciences, Mohammed V University in Rabat, Morocco

**Keywords:** Prevalence, Respiratory viruses, Acute respiratory infections, Morocco

## Abstract

**Background:**

Numerous microorganisms are linked to acute respiratory infections, with increasing focus on viruses as significant pathogens, particularly following the emergence of severe acute respiratory infections. we aimed to evaluate the prevalence of respiratory viruses in patients with acute respiratory infections in different regions of Morocco.

**Materials and Methods:**

Our study was conducted in accordance with the methodological criteria of the preferred reporting items for systematic reviews and meta-analyses (PRISMA). We systematically reviewed studies having using databases of PubMed, Scopus, ScienceDirect, and Web of Science between 2000 and 2023. The protocol of the review was registered in the PROSPERO register (CRD42023372751). Twenty-three studies were included in the review.

**Results:**

The prevalence of pandemic influenza A(H1N1)2009 varied widely, ranging from 8% to 96%, with almost all studies reporting proportions exceeding 30%. Seasonal influenza had a prevalence ranging from 0.88% to 17%. Among children, four studies examined Respiratory Syncytial Virus prevalence, estimating rates between 18% and 36.47%. Additionally, four studies assessed Respiratory Syncytial Virus prevalence across all age groups, reporting rates from 12% to 53.8%. Three studies found Rhinovirus prevalence in children exceeding 50%, while six studies investigating populations of all ages reported rates from 5.8% to 38%.

**Conclusions:**

This review suggests that Pandemic and Seasonal Influenza, Respiratory Syncytial Virus and Rhinovirus have a considerable prevalence in the samples studied in the different cities of the Kingdom of Morocco.

## Introduction

Respiratory infections in the upper and lower airways pose a significant challenge to public health (Yousif and Khaleq, 2006; Ujunwa *et al.*, 2014). It is widely acknowledged as a primary reason for global hospital admissions, and it carries a substantial burden of illness and death, especially in less developed regions (Koch *et al.*, 2003; Sharma *et al.*, 2013), While in the majority of cases, the exact causative agent remains unidentified, empirical antimicrobial treatment is frequently administered and often yields positive outcomes (Remolina *et al.*, 2015). Many microorganisms are associated with acute respiratory infection, and now attention is turning to the importance of viruses as pathogens, especially after emergence of severe acute respiratory syndrome (SARS), avian influenza A (H5N1) virus, and the 2009 pandemic influenza A (H1N1) (Ruuskanen *et al.*, 2011).

The increased risk of acute respiratory infection was most pronounced among young children, the elderly, the chronically ill, and immunocompromised persons (Hodinka, 2016). Effective infection control measures require a good understanding of how pathogens are transmitted (Goldmann, 2000). Acute Respiratory Infections are the leading cause of death among children under five years of age and are responsible for 19% of deaths and around 8% of all disabilities (Shann *et al.*, 1999). Practically, 13% of deaths reported in Moroccan children under five years old in 2012 were caused by Acute Respiratory Infections (World Health Organisation, 2016).

Approximately 80% of respiratory infections are attributed to viruses (Nair *et al.*, 2010). leading the identification of over 200 distinct virus types as causative agents for acute respiratory infections. Among these, respiratory syncytial virus (RSV), influenza viruses, human metapneumovirus (Hmpv), parainfluenza viruses, adenoviruses (AdV), rhinoviruses (RV), coronaviruses (CoV), and bocaviruses (BoV) are the most encountered (Korsun *et al.*, 2017).

New respiratory viruses - such as CoV, Hmpv, and human BoV have been discovered during the past decade (Ruuskanen *et al.*, 2011).

In our current systematic review, we aimed to evaluate the prevalence of respiratory viruses in patients with acute respiratory infections in different regions of Morocco. This review focuses on hospital-based studies to ensure a comprehensive analysis of severe clinical outcomes, as these settings provide detailed data on the most critical cases requiring medical intervention.

## Materials and Methods

This systematic review was conducted in accordance with the methodological criteria of the preferred reporting items for systematic reviews and meta-analyses (PRISMA) (Page *et al.*, 2021). The protocol has been previously registered and published (PROSPERO 2023 CRD42023372751 Available from: https://www.crd.york.ac.uk/prospero/display_record.php?ID=CRD42023372751).

### Study search and selection

We systematically reviewed studies haven used databases of PubMed, Scopus, ScienceDirect, Web of Science and Google Scholar between 2000 and 2023.

We searched for the following terms: “Respiratory Tract infection”, “Severe Acute Respiratory Syndrome”, “Severe Acute Respiratory Infection”, “Influenza Human”, Pandemic influenza A(H1N1)2009, Seasonal influenza virus, “Coronavirus Infections”, “Pneumovirus Infections”, “Paramyxoviridae Infections”, “Hantavirus Pulmonary Syndrome”, “Bronchiolitis Viral”, “Respiratory Syncytial Virus Infections”, “Respiratory Syncytial Viruses”, “ SARS Virus”, “ SARSCov2 “, “Middle East Respiratory Syndrome Coronavirus”, “Coronavirus”, “COVID-19”, “Rhinovirus”, Adenovirus, “Human metapneumovirus”, “Parainfluenza virus”, “Bocavirus”, “Enterovirus”, “Orthomyxoviridae”, “Serotype”, “Subtype”, “Genotype”, “Morocco”. Additionally, we manually searched the reference lists of relevant articles.

Studies that estimated the prevalence of viruses as significant pathogens in patients with influenza-like illness (ILI) and severe acute respiratory infections (SARI) were included.

Two authors (KA and EHO) independently verified titles and abstracts to identify the to-be included studies. Any disagreements regarding inclusion were resolved through discussion with a third researcher (BMA). Complete articles of the potential studies were downloaded for a more detailed evaluation and the list of references in all relevant articles were examined for additional documents as well as for the citing papers.

### Inclusion and exclusion criteria

Only published articles were included in the systematic review. Only articles in English or French, published between 2000 and 2023, with a study population focused on respiratory specimens\Oro-pharyngeal and naso-pharyngeal swabs from outpatients presenting with ILI and patients presenting with SARI at hospitals or syndromic surveillance for ILI and acute respiratory infection were selected. Articles on clinical cases, cases series study, reviews, meta-analyses, cost-effectiveness studies and articles with a lack of information regarding prevalence of circulating viral respiratory pathogens in Morocco were excluded. (See Table 1)

**Table 1:** Eligibility criteria according to the PICOS framework

PICOS items	Inclusion and exclusion criteria
**Population**	Patients or respiratory specimens / Oro-pharyngeal and naso-pharyngeal swabs from outpatients presenting with influenza-like illness (ILI). Patients presenting with severe acute respiratory illness (SARI) at hospitals or syndromic surveillance for influenza like illness (ILI) and acute respiratory infection (ARI).

**Intervention**	Studies that estimated the prevalence of circulating viral respiratory pathogens including emergence of novel zoonotic influenza viruses in Morocco between 2000 and 2023. Studies that investigated the serotypes of circulating viral respiratory pathogens.

**Comparison**	Studies without a comparison group.

**Outcome**	Studies to be eligible for inclusion should include the prevalence type of circulating virus/virus types/subtypes detected/pathogens investigated, prevalence by age, prevalence by gender, and prevalence by seasonality.

**Study design**	We included prevalence studies.

**PICOS:** Population, Intervention, Comparison, Outcome, and Study design.

No restrictions were made on the language of publication.

### Data extraction

The data extracted from the identified documents were the following: Location and period (years), study design, Sample (Specimen), Case ascertainment, Methods used for virus detection or microbiological techniques, sample types or specimen, prevalence by type of circulating virus/virus types/subtypes detected/pathogens investigated, prevalence by age, prevalence by gender, and prevalence by seasonality.

### Risk of bias in individual studies

Bias risk assessment was conducted by two authors independently (KA, BMA) through the use of JBI Critical Appraisal tool for prevalence studies (Munn *et al.*, 2017). The agreement between the two examiners results was analysed by the Kappa statistic coefficient (κ).

## Results

### Study selection

A total of 442 articles were found from the search. After screening of titles and summaries, 35 articles were selected for full-text assessment. Twenty-three (n=23) articles were included in the qualitative synthesis of the systematic review.

### Study characteristics

A total of Twenty-one (n=21) studies were conducted from 2011 through 2023 among a total of 22634 Moroccan suffering from upper respiratory tract infections, influenza-like illness, lower tract respiratory infection, or pneumonia. By locations: National Institute of Hygiene (n=2), Casablanca (n=1), Rabat (n=8), Rabat et casa (n=1), Marrakech (n=4), Agadir (n=1), Clinic and hospital in Morocco (n=1), (n=1) Eight public ambulatory and hospital sentinel sites: Rabat, Tangiers, Fez, Marrakech, Meknes, Beni Mellal, Oujda and Agadir and the private ambulatory network of medical practitioners, (n=1) Eight public sector general hospitals located in all the eight regions in the country, (n=1) sentinel hospitals from Eight regions distributed nationwide, (n=1) in eight public general hospitals located in the country’s main regions, and seven regional sites (n=1).

**Figure 1:**
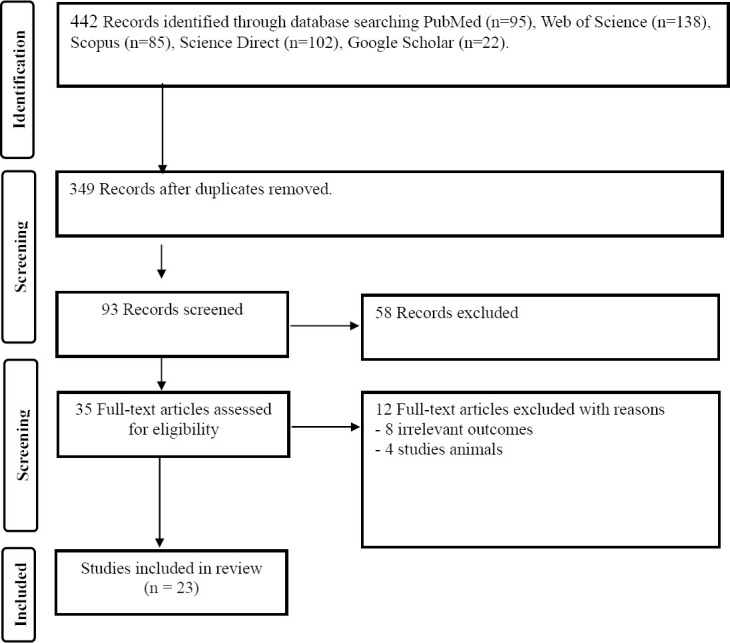
Flow chart of the selected studies in the review.

All studies were transversal in design. Sample size varied from 89 to 3937 individuals. According to the age of the target population of the selected studies, (n=15) studies targeted all ages combined (children and adult), (n=5) studies focused on the children population (under 5 years old) and (n=3) studies focused on the children population (under 15 years old).

Types of samples included were Oro-pharyngeal and naso-pharyngeal swabs in (n=5), Nasopharyngeal aspirates (NPAs) for identification of respiratory viruses in (n=15), Nasopharyngeal secretions and respiratory specimens in (n=1), Blood serum (n=1), Tracheal secretion (n=1), The respiratory secretions were collected from the pediatric inpatients using flocked nasopharyngeal swabs immersed in a universal transport medium (UTM) (upper respiratory tract) or bronchial aspiration and bronchoalveolar lavage (lower respiratory tract) in (n=1).

The prevalence of viruses was investigated in all (n=23) studies. Distinct methods of detection were used in (n=21) studies, RT-PCR (n=15), (n=1) Real-time monoplex reverse-transcription polymerase chain reaction method, (n=1) qRt-PCR and the SuperScript III Platinum® One-Step qRT-PCR System, (n=2) Film Array® instrument (BioMérieux, Marcy-l’Étoile, France) with the Film Array® Respiratory Panel (FA-RP) for the detection of respiratory pathogen, (n=1) using Delta-Swab ViCUM®, (n=1) The multiplex RespiFinder® SMART 22 FAST, (n=1) Multiplex real-time PCR (one step reverse transcription) by using Kit FTD Respiratory Pathogens 21 plus (Fast Track Diagnostics Luxembourg©) which allows the detection of 21 pathogens (viruses and bacteria) and (n=1) Multiplexed nucleic acid test intended for the simultaneous detection and differentiation of 22 respiratory pathogens. The results of the characteristics of the studies are presented in (Table 2).

**Table 2:** Characteristics of studies

Studies	Location and period	Sample	Case ascertainment	Methods Used for Virus Detection/ Microbiological techniques	Sample Types Specimen	Study design
**Barakat *et al* (2011)**	National Institute of Hygiene the Epidemiology Department at the Ministry of Health private Center 2007-2009	3102	ILI and SARI / The virological surveillance system	RT-PCR	Oro-pharyngeal and naso-pharyngeal swabs	A transversal-study
**Lahlou *et al* (2011) 2**	From June 12 to December 24, 2009. Mohammed V Military Training Hospital, Rabat	594	Outpatients	RT-PCR	Naso-pharyngeal swabs	A transversal-study
**Barakat *et al* (2012)**	2009-2010 136 Private clinic and hospital	3937	ILI at private and public clinics	RT-PCR	Oro-pharyngeal and naso-pharyngeal swabs	A transversal-study
**El Rhaffouli (2013)**	From March to April 2011 Rabat Meknes	300 Rabat 200 Meknes	Blood donors	HI^ɬ^ And PCR	Blood serum	A transversal-study
**Anga *et al* (2013)**	From 07 May, 2009 to 17 May 2010. Institute Pasteur of Morocco Casablanca	1347	Patients with clinical evidence ILI	RT-PCR	Nasal/ Nasopharyngeal swab/Broncho Alveolar Lavage (BAL)	A transversal-study
**El Rhaffouli (2014)**	Rabat Casa 2009-2011	1183	Surveillance of influenza A(H1N1)pdm09	RT-PCR	Naso-pharyngeal swabs	A transversal-study
**Jroundi *et al* (2014)**	University Hospital Center IBN SINA RABAT	700 children	Children admitted to the hospital	RT-PCR	Naso-pharyngeal swabs	A transversal-study
**Jroundi *et al* (2014)**	November 2010 to December 2011 Children’s Hospital of Rabat, in Morocco’s capital.	689 children	Children (2–59 months) / Hospital / Clinically severe pneumonia definition	RT-PCR	Nasopharyngeal aspirates	A transversal study
**Mestoui *et al* (2014)**	The Fraternal Laboratory of the Royal Gendarmerie in the Rabat region between November 23, 2009 and March 7, 2010.	300	300 clinical samples of nasopharyngeal swabs	Real-time RT-PCR	Nasopharyngeal swabs	A transversal-study
**Jroundi *et al* (2015)**	University Hospital Center IBN SINA RABAT (November 2010 to December 2011	683 children	Paediatric population admitted to the hospital	RT-PCR	Nasopharyngeal aspirate	A transversal-study
**Elfalki *et al* (2016)**	National Institute of Hygiene)	440	Outpatients presenting with influenza- like illness (ILI) and inpatients presenting with severe acute respiratory illness (SARI)	RT-PCR	Oro-pharyngeal and naso-pharyngeal swabs	A transversal-study
**Bimouhen *et al* (2016)**	Respiratory syncytial virus surveillance system in Morocco (2014 and 2016)	1450 specimens	Sentinel-based influenza surveillance / Hospitalized patients and outpatient clinics	Real time RT-PCR	Both nasopharyngeal (NP) and oropharyngeal (OP)	A transversal study
**Annamalay *et al* (2017)**	Clinically severe pneumonia (CSP) to the Hôpital d’Enfants de Rabat (HER)	700 children	Children admitted to HER with CSP	TrueScience® RespiFinder Pathogen Identification Panel	Nasopharyngeal aspirates (NPAs)	A transversal-study
**Marcil *et al* (2018)**	Ibn Sina University Hospital, between October 2015 and August 2016	147 patients	Patients hospitalized for severe acute infection	Multiplex real-time PCR (one step reverse transcription)	Nasopharyngeal (n= 116), bronchoalveolar lavages, (n= 1), bronchial aspiration (n= 1) or PDP (n=3).	A transversal-study
**Daoudi *et al* (2019)**	Hospital Arrazi University Hospital Center Mohammed VI, Marrakech Morocco) from January to December 2018	338 patients	Patients hospitalized for lower respiratory tract infection	RT-PCR	Nasopharyngeal specimen	A transversal-study
**Hattoufi *et al* (2020)**	Children’s Hospital of Rabat. The unit of Neonatology between December 1, 2016, and Mai 31, 2018.	86 infants	Infants admitted with respiratory distress isolated or associated with systemic signs.	The multiplex RespiFinder® SMART 22 FAST	Tracheal secretion	A transversal-study
**Ezzine *et al* (2020)**	Epidemiological and virological surveillance of severe acute respiratory infections and influenza like illness 2016/2017 and 2017/2018 seasons	2016/2017 :1359 samples/ 2017/2018 :1263 samples	Eight public ambulatory and hospital sentinel sites: Rabat, Tangiers, Fez, Marrakech, Meknes, Beni Mellal, Oujda and Agadir and the private ambulatory network of medical practitioners.	RT-PCR	Nasal and pharyngeal sampling	A transversal-study
**Ighid *et al* (2020)**	Hassan II regional hospital of Agadir, Morocco, (September 2015 to September 2016)	84 patients	Hospitalized Moroccan Children under 15 Years	RT-PCR	Nasal or nasopharyngeal swabs	A transversal study
**Lamrani Hanchi *et al* (2021)**	A period of 24 months from January 2018 to December 2019	534 children	Hospitalised in paediatric department	RT-PCR	Nasopharyngeal secretions and respiratory specimens	A transversal study
**Lamrani Hanchi *et al* (2022)**	Before COVID-19 from January 2018 to February 2020, and during COVID-19 from March 2020 to December 2021. Mohamed VI Pediatric University Hospital in Marrakech.	586 samples before the COVID-19 period and 316 during COVID-19.	Children under fourteen years of age hospitalized for the management of Severe Acute Respiratory Infection.	RT-PCR	Nasopharyngeal (upper respiratory tract) or bronchial aspiration and bronchoalveolar lavage (lower respiratory tract).	A transversal study
**Regragui *et al* (2022)**	The National Influenza Center at the National Institute of Hygiene in Morocco (September 2018 to March 2019 period)	942 SARI specimens tested among children under 5 years old.	The samples tested were collected from 8 sentinel sites distributed throughout the country.	RT-PCR	Nasopharyngeal swabs.	A transversal-study
**Bimouhen *et al* (2022)**	September 1, 2014, to December 31, 2016	1187 from ILI and 822 from SARI sentinel	Inpatients and outpatients	RT-PCR	Nasopharyngeal (NP) and oropharyngeal (OP) specimens	A transversal-study
**Regragui *et al* (2023)**	From February to July 2021, from seven regional sites in Morocco.	167	-	Multiplex PCR	nasopharyngeal swabs	A transversal study

The evaluation of the quality of the studies was considered “Good” with a mean score of 8.22. The Kappa statistical coefficient was (κ=0.70). The results of the methodological evaluation of the studies are presented in (Table 3).

**Table 3:** Assessment of the methodological quality of included studies using the JBI Critical Appraisal Checklist for studies reporting prevalence data.

Criteria/Study	1	2	3	4	5	6	7	8	9	10	11	12	13	14	15	16	17	18	19	20	21	22	23
1. Was the sample frame appropriate to address the target population?	1	1	1	1	1	1	1	1	1	1	1	1	1	1	1	1	1	1	1	1	1	1	1
2. Were study participants sampled in an appropriate way?	1	1	1	1	1	1	1	1	1	1	1	1	1	1	1	1	1	1	1	1	1	1	1
3. Was the sample size adequate?	1	1	1	1	1	1	1	1	1	1	1	1	1	1	0	1	1	1	1	1	1	1	1
4. Were the study subjects and the setting described in detail?	1	0	1	0	0	0	1	1	1	1	1	1	1	1	0	1	1	0	1	1	1	1	1
5. Was the data analysis conducted with sufficient coverage of the identified sample?	1	1	1	1	1	1	1	1	1	1	1	1	1	1	1	1	1	1	1	1	1	1	0
6. Were valid methods used for the identification of the condition?	1	1	1	1	1	1	1	1	1	1	1	1	1	1	1	1	1	1	1	1	1	1	1
7. Was the condition measured in a standard, reliable way for all participants?	1	1	1	1	1	1	1	1	1	1	1	1	1	1	1	1	1	1	1	1	1	1	1
8. Was there appropriate statistical analysis?	1	1	1	1	1	0	0	1	1	1	1	0	0	0	0	0	1	0	1	1	0	0	1
9. Was the response rate adequate, and if not, was the low response rate managed appropriately?	1	1	1	1	1	1	1	1	1	1	1	1	1	1	1	1	1	1	1	1	1	1	1
**Score**	**9**	**8**	**9**	**8**	**8**	**7**	**8**	**9**	**9**	**9**	**9**	**8**	**8**	**8**	**6**	**8**	**9**	**7**	**9**	**9**	**8**	**8**	**8**

same ranking of studies in table

### Prevalence of circulating viral respiratory pathogens in selected studies

### Prevalence of the seasonal and pandemic influenza A(H1N1)2009 in selected studies

Eight studies focused on pandemic influenza A(H1N1)2009. The prevalence of this type of virus varied between 8% and 96%, the proportion of pandemic influenza A(H1N1)2009 exceeded 30% in almost all studies (Amine *et al.*, 2011; Barakat *et al.*, 2012; El Rhaffouli et al., 2013; Anga *et al.*, 2013; El Rhaffouli *et al.*, 2014; Mestoui *et al.*, 2014; Elfalki *et al.*, 2016; Regragui *et al.*, 2022). In the first study, the prevalence of pandemic influenza A(H1N1)2009 was estimated at 240 (40%) (Amine *et al.*, 2011). In the second study, 1452 tested positive for influenza virus (37%). 1398 (96%) were A(H1N1)pdm09 (Barakat *et al.*, 2012). In the third study conducted in the two cities Rabat and Meknes, the overall seroprevalence of A(H1N1) pdm09 infection was 53% in Meknes. In Rabat, the overall seroprevalence was 67%, (67% versus 53%, p < 0.05) (El Rhaffouli *et al.*, 2013). The fourth study, 489 (36.3%) were positive for A (H1N1)pdm09 and 858 (63.7%) were negative (Anga *et al.*, 2013). The Fifth study: influenza A(H1N1)pdm09 was detected in 373 (31%) of 1183 suspected cases (El Rhaffouli *et al.*, 2014). In the sixth study, pandemic influenza A pH1N1 was detected in 149 cases (49.7%), while seasonal influenza A viruses were detected in only 14 cases (4.6%) (Mestoui *et al.*, 2014). In the seventh study, 38 (8%) was the proportion of A(H1N1) pdm09 explored in the study population (Elfalki *et al.*, 2016). In the eighth study, among all confirmed influenza cases, 68.75% were subtyped as AH1N1pdm09 (Regragui et al., 2022).

Hight studies reported the prevalence of seasonal influenza, which ranged from 0.88 % to 17% (Barakat *et al.*, 2012; Mestoui *et al.*, 2014; Elfalki *et al.*, 2016; Marcil *et al.*, 2018; Daoudi *et al.*, 2019; Hattoufi *et al.*, 2020; Ighid et al., 2020; Lamrani Hanchi *et al.*, 2021). In the first study, 17 cases (1.1%) were seasonal influenza subtype A (A[H1N1]), 4 cases (0.2%) were seasonal influenza subtype H3N2 (A[H3N2]) and 33 cases (2.3%) were seasonal influenza type B (Barakat *et al.*, 2012). In the second study, seasonal influenza A viruses were detected in only 14 cases (4.6%) (Mestoui *et al.*, 2014). In the third study, 135 (31%) cases of influenza B and 25 (6%) cases of influenza A (H3N2) were detected (Elfalki *et al.*, 2016). In the fourth study, influenza virus (A/B) was detected in only 3 cases (0.88%) (Daoudi *et al.*, 2019). In the fifth study, H1N1 influenza virus was detected in (1%) (Hattoufi *et al.*, 2020). In the sixth study, influenza A virus (InfluA) was found in 1.9% of cases (Ighid *et al.*, 2020). In the seventh study, InfluA was revealed in n= 29/340 (8.52%) (Lamrani Hanchi *et al.*, 2021). In the eighth study, 21 (17%) cases flu infections has been shown (Marcil *et al.*, 2018).

Other studies have estimated the prevalence of seasonal influenza. One study mentioned the prevalence of this type of virus in the years 2007-2008 (ILI and SARI=19), influenza A (H1N1) (5/19; 26%) and B (H1N1) (14/19; 74%). 2008-2009 (ILI and SARI=79), influenza A (H1N1) (39/79; 49%), A (H3N2) (30/79; 38%) and B (10/79; 13%) (Barakat *et al.*, 2011). Another investigation showed that Influenza was the most common virus, detected in 612 (30.5%) positive samples (Bimouhen *et al.*, 2022). Among all confirmed influenza cases, 68.75% (77/112), 15.17% (17/112), 16.04% (18/112) were AH1N1pdm09, AH3N2 and B influenza subtypes respectively. Among them, 94.44% (17/18) were influenza B/yamagata subtypes (Regragui *et al.*, 2022).

### Prevalence of the Respiratory Syncytial Virus in selected studies

Nine studies focused on the prevalence of RSV (El Rhaffouli *et al.*, 2014; Jroundi *et al.*, 2014; Bimouhen *et al.*, 2016; Jroundi *et al.*, 2016; Daoudi *et al.*, 2019; Hattoufi *et al.*, 2020; Ighid *et al.*, 2020; Hanchi *et al.*, 2021; Bimouhen *et al.*, 2022). Four studies explored the prevalence in children, which estimated a prevalence ranging from 18% to 36.47%. The first study, which included 700 children, found a proportion of RSV of 18% (Jroundi *et al.*, 2014). The second study, on a sample of 683 children aged 2-59 months, the proportion of RSV was estimated at 124/683 (18.2%) (Jroundi *et al.*, 2016). The third study, on a sample of 86 infants, the proportion of the same RSV (A and B) was 49%) (Hattoufi *et al.*, 2020). The fourth study, conducted in 534 children, RSV was detected in (n=124/340) (Hanchi *et al.*, 2021).

Furthermore, five other studies measured the proportion of RSV in samples of all ages, ranging from 12% to 53.8%. RSV was detected in 18.4% (267/1450) (Bimouhen *et al.*, 2016). Another study highlighted RSV only in 29 (8.57%) (Daoudi *et al.*, 2019). On the other hand, the results of another investigation showed a predominance of RSV in 53.8% of cases (El Rhaffouli *et al.*, 2014). While a study, explained that RSV was present only in (n = 359, 17.9%) of the study population (Bimouhen *et al.*, 2022). 15 (12 %) cases of RSV A/B (Marcil *et al.*, 2018).

### Prevalence of the Rhinovirus in selected studies

Three studies have reported a prevalence of RV in children that exceeds 50% (Jroundi *et al.*, 2014; Hattoufi *et al.*, 2020; Hanchi *et al.*, 2021). The first study, in a sample of 700 children, RV was detected in 53% (Jroundi *et al.*, 2014). The second study, on a sample of 86 infants RV is revealed in (21%) (Hattoufi *et al.*, 2020). The third study, on 534 children HRV was present in (n=201/340 59.11%) of cases (Hanchi *et al.*, 2021).

Six other studies explored the prevalence of the same virus in samples of populations of all ages, ranging from 5.8% to 38% (Annamalay *et al.*, 2017; Marcil *et al.*, 2018; Daoudi *et al.*, 2019; Ighid *et al.*, 2020; Bimouhen *et al.*, 2022; Regragui *et al.*, 2023). The first study, HRV was present in 89 (26.33%) (Daoudi *et al.*, 2019). The second study, RV was detected in 5.8% of patients (Ighid *et al.*, 2020). The third study, HRV in (n = 263, 13.1%) (Bimouhen *et al.*, 2022).

One study focused only on RV and serotypes. Human RV was reported in 53% of cases. The proportion RV-A was 60 (38.2%), RV-B was 8 (5.1%) and RV-C was 89 (56.7%) (Annamalay *et al.*, 2017). 34 (28%) cases of RV infections (Marcil *et al.*, 2018). The main detected pathogens were typed as Human RV (38%) *(Regragui et al.*, 2023).

### Prevalence of the Adenovirus in selected studies

Two studies have reported the prevalence of RV in children. The first study of 700 children found AdV in (17%) (Jroundi *et al.*, 2014). In the second study, which involved 86 infants, AdV was detected in 2% of them (Hattoufi *et al.*, 2020). In contrast, four other studies have examined the proportion of ADV in samples of all ages. In the first study, ADV was found in only 3 (0.88%) of the cases (Daoudi et al., 2019). In the second study, AdV was found in 1.9% (Ighid *et al.*, 2020). In the third study, HAdV was present in (n = 124, 6.2%) (Bimouhen *et al.*, 2022). seven (6%) cases of AdV (Marcil *et al.*, 2018).

### Prevalence of Human metapneumovirus in selected studies

Four studies have determined the proportion of Hmpv in children, which varies between 5% and 9.1%. In the first study, the prevalence of HMPV was estimated at 9.1% (Jroundi *et al.*, 2014). The second study, HMPV was found in 61/683 (8.9%) of the study population (Jroundi *et al.*, 2016). The third study, metapneumovirus was present in only 5% (Hattoufi *et al.*, 2020). The fourth study, metapneumovirus was found in (n=27/340=7.94%) (Koch *et al.*, 2003). However, the prevalence in samples of all ages, three studies have demonstrated the proportion of the said virus. In the first study, HMP was found in only 11 (3.25%) (Daoudi *et al.*, 2019). The second study showed a proportion of (n = 74, 3.7%) (Bimouhen *et al.*, 2022). 14 (11%) cases of Hmpv A/B (Marcil *et al.*, 2018).

### Prevalence of Coronavirus in selected studies

Concerning coronavirus, the proportion of CoV 229E is estimated at (11%) in a study conducted in children (Hattoufi *et al.*, 2020). Nevertheless, five other studies reported a prevalence varying between 2.07% and 37% in the population of all ages ( El Rhaffouli *et al.*, 2014; Marcil *et al.*, 2018; Daoudi *et al.*, 2019; Bimouhen *et al.*, 2022; Regrarui *et al.*, 2023). To this end, the first study, coronavirus (43/229E/NL 63 only) was found in 7 (2.07%) of the cases (Daoudi *et al.*, 2019). The second study, coronavirus (CoV) was detected in 5.8% of patients (El Rhaffouli *et al.*, 2014). The third study, HCoV was present in (n = 94, 4.7%) (Bimouhen *et al.*, 2022). Seven (6%) cases of coronavirus including two coronavirus 229E, two coronavirus HKU, two coronavirus OC43 and one coronavirus NL63 (Marcil *et al.*, 2018). The main detected pathogens were Human Coronavirus OC43 (37%) (Regrarui *et al.*, 2023).

### Prevalence of Parainfluenza virus in selected studies

In relation to parainfluenza virus, the proportion of parainfluenza virus type 4 was estimated to be 2% in one study (Hattoufi *et al.*, 2020) and 10.29% in another study conducted among children (Lamrani Hanchi *et al.*, 2021). On the other hand, the prevalence of the same virus was between 2.66% and 12% in five investigations carried out in samples of all ages. The first study, Parainfluenza (1-4) was found in only 9 (2.66%) cases (Daoudi *et al.*, 2019). The second investigation, nucleic acids of Para Influenza (PIV) were found in 3.8% of cases (Ighid *et al.*, 2020). The third investigation, PIVs were revealed in (n = 107, 5.3%) (Bimouhen *et al.*, 2022). 11 (9%) cases parainfluenza virus including eight parainfluenza 3, two parainfluenza 4, one parainfluenza 1 (Marcil et al., 2018). The main detected pathogens were typed as Parainfluenza3 (HPIV3) (12%) (Regrarui *et al.*, 2023).

### Prevalence of others circulating viral respiratory pathogens in selected studies

 Only one study specified the prevalence of BoV and enterovirus (ETV), at 4% and 7% respectively (Marcil *et* *al*., 2018).

### Prevalence of circulating viral respiratory pathogens by age

### Prevalence of influenza by age

Overall, the prevalence of influenza by age was revealed in three studies (Barakat *et al.*, 2011; Elfalki *et al.*, 2016; Regragui *et al.*, 2022). Two studies mentioned prevalence by age and by ILI or SARI (Barakat *et al.*, 2011; Elfalki *et al.*, 2016), and a third study focused only on the prevalence of influenza in age categories among children (Regragui *et al.*, 2022).

The first investigation demonstrated that among ILI patients, the highest proportion of laboratory-confirmed influenza occurred in children less than 5 years of age (3/169; 2% during 2007–2008 and 23/271; 9% during 2008–2009) and patients 25–59 years of age (8/440; 2% during 2007–2009 and 21/483; 4% during 2008–2009). All SARI patients with influenza were less than 14 years of age (Barakat *et al.*, 2011). concerning the second study, Influenza prevalence among patients with ILI and SARI by age: 0-23 months: ILI 5 (1.8) / SARI 71 (42.5); 2-5 years: ILI 19 (7.0)/SARI 13 (7.8); 6-15 years: ILI 50 (18.3)/SARI 14 (8.4); 16-49 years: ILI 112 (41.0)/ SARI 28 (16.8); 50-64 years: ILI 59 (21.6)/SARI 17 (10.2); >65 years: ILI 16 (5.9)/SARI 15 (9.0) (Elfalki *et al.*, 2016). Compared to the third study, the detection rate for influenza was higher among the children between 6 and 23 months (47.32% ; 53/112), followed by those aged between 2 and 5 years (30.35% ; 34/112) and then Children under 6 months (22.32% ; 25/112) (Regragui *et al.*, 2022).

### Prevalence of pandemic influenza A(H1N1)2009 by age

Three studies focused on the prevalence of pandemic influenza A(H1N1)2009 according to the studies included in this review (Barakat *et al.*, 2012; El Rhaffouli *et al.*, 2013; Mestoui *et al.*, 2014). The first showed that the highest percentages of positive A(H1N1)pdm09 cases among ILI (490 of 911; 54%) and SARI (73 of 220; 33%) cases were observed in the 5–14-year age group (Barakat *et al.*, 2012). The second carried out in Meknes revealed, the seroprevalence of pandemic influenza A(H1N1)2009 antibodies, was 57, 57, 49 and 64% in subjects under 25, 25-40, 40-50 and over 50 years old, respectively. Therefore, In Rabat, the seroprevalence of A(H1N1)pdm09 antibodies was 83, 56, 65 and 72% in subjects under 25, 25-40, 40-50 and over 50 years old, respectively (El Rhaffouli *et al.*, 2013). The third confirmed that the distribution of pH1N1 infected cases according to age clearly showed that children between 5 and 14 years are the most affected by pH1N1: 82⁄149 (55%, p = 0, OR = 5.25, CI 95% 2.68-10.27). Of particular interest, 25 children less than 4 years were confirmed pH1N1 positive representing 16.78% of total positive cases (Mestoui *et al.*, 2014).

### Prevalence of Respiratory syncytial virus by age

The prevalence of RSV according to age was recorded in four studies (Bimouhen *et al.*, 2016; Marcil *et al.*, 2018; Ighid *et al.*, 2020; Bimouhen *et al.*, 2022) and another study compared the prevalence of RSV and hMPV by age (Jroundi *et al.*, 2016).

0-6 month : 121 (45)/ 7-23 month : 52 (19)/ 2-4 year : 31 (12)/ 5-14 year : 7 (3)/ 15-49 year : 25 (9)/ 50-64 year : 16 (6)/ >65 year : 7 (3)/Unknown : 8 (3) (Bimouhen *et al.*, 2016). The most infected patients were under two years old. Results from this patients group showed a predominance of RSV with 50% of positive cases against 3.8% in children of 3 to 5 years and null in the last group (6 to 15 years) (Ighid et al., 2020). SARI/ <5 years, RSV 228 (42.4). >5 years, RSV 10 (3.7) (Bimouhen *et al.*, 2022). Respiratory samples collected from pediatric patients versus respiratory samples from adult patients. The correlation between the presence of the viruses and age group, objectified statistically significant differences with regard to the RSV. indeed, we found 14 cases of RSV (93%) at the pediatric patients and only one case (7%) in adult patients (p=0.04) *(Marcil et al.*, 2018). Age, months, median (IQR), RSV cases (n = 124): 10 (4–24) / hMPV cases (n = 61): 15 (7–25)/P value: 0·072. Group age <12 months RSV cases (n = 124) : 65/124 (52·4) / hMPV cases (n = 61) : 25/61 (41·0) / P value: 0·143 (Jroundi et al., 2016).

### Prevalence of others virus by age

A study treating children with RV-A and RV-C hospitalized for clinically severe pneumonia in Rabat revealed a mean age of 16.5 for RV-A and 24.0 for RV-C (Annamalay *et al.*, 2017).

### Prevalence of viruses by gender

### Prevalence of Influenza and pandemic influenza A(H1N1)2009 by sexe

One study focused on estimating the prevalence of influenza in ILI patients compared with SARI patients according to gender. This study showed a slight predominance of influenza in female ILI patients 155 (56.7) compared with SARI patients 81 (48.5) (Elfalki *et al.*, 2016). Another study revealed a male predominance in all influenza confirmed cases (Male 71 (63.39)/Female 41 (36.60)) (Regragui *et al.*, 2022).

Concerning the 2009 A(H1N1) Influenza Pandemic, a study revealed that more than the majority of positive cases are male, 149/240 (62%) (Amine *et al.*, 2011). Among the A(H1N1)pdm09-positive cases, there were 716 (51%) males and 682 (49%) females (Barakat *et al.*, 2012). Influenza A(H1N1) pdm09. In Meknes, seroprevalence among men was higher than among women, but the difference was not significant (p = 0.81). Rabat, Seroprevalence was higher in men than in women but the difference was not statistically significant (p = 0.65) (El Rhaffouli *et al.*, 2013). Of the 149 pH1N1 positive cases, 81 (54.40%) were women and 68 (45.60%) were men (M⁄F ratio 0.9:1) (Mestoui *et al.*, 2014). A(H1N1)pdm09, Male 230 (47.0%)/ Female 258 (52.8%) (p=0.231) (Anga *et al.*, 2013).

### Prevalence of Respiratory syncytial virus by sexe.

Concerning RSV according to sex, the results of a study revealed a non-significant difference in prevalence between sexes: Female P/N: 129 (17)/628 (83). Male P/N: 138 (20)/555 (80) P value 0.159 (Bimouhen et al., 2016).

For children, the rate of positivity in boys was similar to girls (n= 225/310, 72.6%; n=162/224, 72.3%; p= 0.947) (Lamrani Hanchi *et al.*, 2021). *Sex, female* RSV cases (n = 124): 46/124 (37·1)/hMPV cases (n = 61): 26/61 (42·6)/ P value: 0·469 (Jroundi *et al.*, 2016).

### Prevalence of others circulating viral respiratory pathogens by sexe

The study evaluating the circulation of RV showed that the prevalence by sex was : Gender Male RV-A (n=60) 37 (61.7%)/ RV-C (n=89) 59 (66.3%) (Annamalay *et al.*, 2017).

### Prevalence of viruses by seasons

### Prevalence of Influenza season by seasons

During the 2007–2008 influenza season, influenza viruses were isolated from week 49/07 (December 3–9, 2007) to week 13/08 (March 24–30, 2008) and peaked during weeks 3/08 and 4/08 (January 14–27, 2008). During the 2008–2009 influenza season, influenza viruses were detected from week 43/08 (October 20–26, 2008) to week 16/09 (April 13–19, 2009) and peaked during weeks 1/09 and 2/09 (December 29, 2008–January 11, 2009) (Barakat *et al.*, 2011). All influenza Positives: First Quarter 90 (80.35); Second Quarter 9 (8.03); Third Quarter 0 (0); Fourth Quarter 13(11.60) (Regragui *et al.*, 2022).

### Prevalence of pandemic influenza A(H1N1)2009 by seasons

At the beginning of the study on November 23rd 2009, 23 pH1N1 positive cases (15.4%) were registered the week 48/2009. Number of cases increased and reached the peak during the week 50/2009 with 58 pH1N1 positive cases (38.9%) and then decreased progressively (Mestoui *et al.*, 2014). Pandemic influenza A(H1N1)2009. A rapid increase in the number of confirmed cases was observed with a peak in weeks 47 to 49 (16 November to 6 December 2009): 172 cases were detected during this time (Amine *et al.*, 2011). pandemic A(H1N1) pdm09 virus. The transmission was began in June, reached its peak in November and declined dramatically subsequently (Anga *et al.*, 2013).

### Prevalence of Respiratory syncytial virus by seasons

Viral activity persists consistently throughout the year, yet there is concurrent circulation of influenza and RSV viruses occurring between November and April, with the highest incidence observed during the months of December through March (Bimouhen *et al.*, 2022).

Another study confirmed the predominance of RSV in the First Quarter, with a proportion of 86% (Bimouhen *et al.*, 2016). RSV infection showed the most pronounced seasonality trends. Indeed, it typically exhibits a clear seasonality pattern with obvious peak in January and lowest activity in September and November. Concerning AdV and RV viruses, peaks were in December and November respectively, while the low number of cases infection by the other respiratory pathogens unable detection of any seasonality (Ighid *et al.*, 2020).

### Prevalence of others circulating viral respiratory pathogens by seasons

RV in autumn and lowest in winter (42.7% vs. 13.4%), RV-A spring (43.3%) and autumn (31.7%)/ summer (15.0%) and winter (10.0%), RV-C autumn (49.4%) and spring (11.2%) (Annamalay *et al.*, 2017). HAdVs and HRV circulated throughout the year with peaks during the winter months. Other viruses often circulated during the cold season with sporadic cases throughout the year (Bimouhen *et al.*, 2022).

With regard to the seasonal distribution of the 122 viruses, a peak was observed in winter: 62% (76/122). In spring, we found 15% (18/122) of viral infections, in summer 15% (18/122) and autumn 8% (10/122). Regarding the distribution of respiratory viruses over the year, influenza virus, RSV and Hmpv showed a clear seasonal pattern, with a marked increase in positivity rate throughout the winter months, while ETV, AdV, RV and parainfluenza 3 prevalence remained relatively stable over the year and did not have marked seasonal variation. We also compared the viral distribution according to the seasons and we belonged to the statistically significant differences for the RSV and the metapneumovirus: indeed, the infections with RSV were diagnosed between mid-January and at the end of April with a peak in February: 13 cases detected in winter and one case in spring (p= 0.008). The Hmpv circulated exclusively in winter (p=0.002) (Marcil *et al.*, 2018). The results of the prevalence of circulating virus in Morocco in selected study are presented in (Table 4).

**Table 4:** Circulating viral respiratory pathogens in Morocco.

Authors (years)	Seasonality N (%)	Prevalence N (%)	Type of circulating virus Virus Types/ Subtypes Detected/ Pathogens investigated	Age affected N (%)	Gender affected N (%)
A. Barakat (2011)	- 2007–2008 influenza season viruses: From week 49/07 (December 3–9, 2007) to week 13/08 (March 24–30, 2008) / peaked during weeks 3/08 and 4/08 (January 14–27, 2008). - 2008–2009 influenza season: From week 43/08 (October 20–26, 2008) to week 16/09 (April 13–19, 2009) / peaked during weeks 1/09 and 2/09 (December 29, 2008–January 11, 2009).	- Laboratory-confirmed influenza: 98/3102 patients, soit (3%). - Laboratory-confirmed Influenza ILI: 85/98 (87%) - Laboratory-confirmed Influenza SARI: 13/98 (13%) - 2007–2008 (ILI and SARI=19): Influenza A (H1N1) (5/19; 26%) and B (H1N1) (14/19; 74%). - 2008–2009 (ILI and SARI=79): Influenza A (H1N1) (39/79; 49%), A (H3N2) (30/79; 38%) and B (10/79; 13%). Tested positive for other viruses (n=29): -RSV: (25/29; 86%) -Adenovirus: (2/29; 7%) -Parainfluenza viruses: (2/29; 7%)		ILI: 2007-2008/2008-2009 - < 5 years: 3/169 (1.78) / 23/271 (8,49) -5-14 years: 2/136 (1,47) / 10/177 (5,65) -15-24 years: 1/167 (0,59) / 12/216 (5,56) -25-59 years: 8/440 (1.82) / 21/483 (4,35) ->60 years: 1/85 (1,18) / 4/105 (3.81) SARI: 2007-2008/2008-2009 -< 5 years: 1/345 (0,29)) / 8/351 (2,28) -5-14 years: 3/27 (11.11) / 1/38 (2,63) -15-24 years: 0/10 (0.00) / 0/22 (0.00) -25-59 years: 0/20 (0.00) / 0/26 (0.00) -> 60 years: 0/1 (0.00) / 0/13 (0.00)	-
A. Lahlou *et al* (2011)	There was a rapid increase in confirmed cases, peaking during weeks 47 to 49 (from November 16th to December 6th, 2009), with a total of 172 cases detected during this period.	Pandemic influenza A(H1N1)2009: 240 (40%)		Mean Age and standard deviation -Total: 28±16 years -Negative patients: 31±17 -Positive patients: 23±14 -(P value: 0.004)	Patients with influenza-like illness A(H1N1)2009 -Male 149/240 (62%) (Positive) -Male 200/354 (56%) (Negative) -P value 0.175
A. Barakat 2012	-	1452 tested positive for influenza virus (37%) -A(H1N1)pdm09 : 1398 (96%) -Seasonal influenza A virus subtype (A[H1N1]): 17 (1.1%) -Influenza A virus subtype H3N2 (A[H3N2]): 4 (0.2%) -Influenza B virus: 33 (2.3%)		A(H1N1)pdm09 (5–14-year age group): - ILI (490 of 911; 54%) -SARI (73 of 220; 33%)	A(H1N1)pdm09-positive cases -Males: 716 (51%) -Females: 682 (49%)
Hi. El Rhaffouli (2013)	-	A (H1N1) pdm09 infection Meknes: 53%. Rabat: 67%, (67% versus 53%, p < 0.05)	The seroprevalence of pre-existing antibodies to influenza A (H1N1) pdm09 in blood donor serums from 2007 is 7.3%.	In Meknes, the seroprevalence A(H1N1)pdm09: -Under 25: 57 % -25-40: 57 % -40-50: 49 % -Over 50 years: 64 % In Rabat, the seroprevalence of A(H1N1)pdm09: -Under 25: 83 % -25-40: 56 % -40-50: 65 % -Over 50 years: 72 %	- Meknes, seroprevalence among men was higher than among women (p = 0.81). - Rabat, Seroprevalence was higher in men than in women (p = 0.65).
L. Anga *et al* (2013)	The transmission was began in June, reached its peak in November and declined dramatically subsequently.	A(H1N1)pdm09 -Positive: 489 (36.3%) -Negative 858 (63.7%)		-15-44 years (46.4%) -5-14 years (32.3%) -Group > 65 years (1.7%) -(p<0.001)	-Male 230 (47.0%) -Female 258 (52.8%) -(p=0.231)
H. El Rhaffouli (2014)	-	The influenza A(H1N1)pdm09: 373 (31 %)		-	-
I. Jroundi (2014)	_	Positivity detection: 91.8 %	-Rhinovirus: (53 %) -Virus respiratoire syncytial: (18 %) -Adenovirus: (17 %), -HMPV^¥^: 9,1%	< 5 years	-Male 449/700 -Female 251/700
I. Jroundi *et al* (2014)	Viruses isolated -Human metapneumovirus Good prognosis: 33 (6.76) Poor prognosis: 27 (15.08) OR (IC)/P value: 2.48 (1.44–4.29)/0.007 -Rhinovirus Good prognosis: 282 (56.97) Poor prognosis: 72 (40.22) OR (IC)/P value: 0.50 (0.35–0.72)/0.001 -Coronavirus Good prognosis: 41 (8.28) Poor prognosis: 13 (7.26) OR (IC)/P value: 0.87 (0.45–1.66) 0.66	-Influenza virus Good prognosis: 14 (2.83) Poor prognosis: 10 (5.60) OR (IC)/P value: 2.03 (0.88–4.66) 0.08 -Parainfluenza virus Good prognosis: 92 (24.58) Poor prognosis: 44 (18.60) OR (IC)/P value: 1.43 (0.95–2.15) 0.08 -Respiratory syncytial virus Good prognosis: 93 (18.80) Poor prognosis: 32 (17.88) OR (IC)/P value: 0.94 (0.60–1.47) 0.78 -Adenovirus Good prognosis: 86 (17.37) Poor prognosis: 30 (16.76) OR (IC)/P value: 0.96 (0.61–1.51) 0.85		-Age, months, mean ± SD Good prognosis 23.40±14.80 Poor prognosis 16.00±12.10 P value = 0.001 -Age < 12 months Good prognosis 123 (24.50) Poor prognosis 89 (47.59) OR 2.80 IC (1.97-2.98) P value = 0.001	-Female sex Good prognosis: 187 (37.25) Poor prognosis: 62 (33.16) OR (IC)/P value: 0.84 (0.59-1.19)/0.32.
Mestoui *et al* (2014)	- November 23rd, 2009, 23 pH1N1 positive cases (15.4%) / the week 48/2009. - The peak / the week 50/2009 pH1N1 positive cases (38.9%)	Week 48/2009 to week 9/2010 - Pandemic influenza A pH1N1: 149 cases (49.7%) - Seasonal influenza A: 14 cases (4.6%)		Distribution of pH1N1 cases according to the age: - < 4: 25 (16.78) - 5-14: 82 (55.03) - 15- 21: 15 (10.07) - 22- 40: 21 (14.09) - 41- 60: 5 (3.36) - > 60: 1 (0.67) - Children between 5 and 14 years are the most affected by pH1N1: 82⁄149 (55%, p = 0, OR = 5.25, CI 95% 2.68-10.27). - 25 children less than 4 years were confirmed pH1N1 positive representing 16.78% of total positive cases.	149 pH1N1 positive cases, -WOMEN: 81 (54.40%) -MEN: 68 (45.60%) -M⁄F ratio: 0.9:1
I. Jroundi *et al* (2015)	Influenza season	- HMPV ^¥^: 61/683 (8·9%) - RSV ^˩^: 124/683 (18·2%)		Age, months, median (IQR) -RSV cases (n = 124): 10 (4–24) -hMPV cases (n = 61): 15 (7–25) -P value: 0·072 Group age <12 months -RSV cases (n = 124): 65/124 (52·4) -hMPV cases (n = 61): 25/61 (41·0) -P value: 0·143	Sex, female -RSV cases (n = 124): 46/124 (37·1) -hMPV cases (n = 61): 26/61 (42·6) -P value: 0·469
ELFALKI (2016)	Influenza season	- Influenza B: 135 (31 %) - A(H1N1) pdm09: 38 (8 %) - A (H3N2): 25 (6 %)		Influenza prevalence among patients with ILI and SARI by age ILI/SARI -0-23 months: 5(1.8)/71 (42.5) -2-5 years: 19(7.0)/13 (7.8) -6-15 years: 50 (18.3)/14 (8.4) -16-49 years: 112(41.0)/ 28 (16.8) -50-64 years: 59 (21.6)/17 (10.2) ->65 years:16 (5.9)/15 (9.0)	Influenza prevalence among patients with ILI and SARI by sexe Female: -ILI: 155 (56.7) -SARI: 81 (48.5
A. Bimouhen (2016)	- First Quarter: 230 (86) - Second Quarter: 1 (0) - Third Quarter: 0 (0) - Fourth Quarter: 36 (13)	Respiratory syncytial virus (RSV) : (18.4%) (267/1450)		- 0-6 month: 121 (45) - 7-23 month: 52 (19) - 2-4 year: 31 (12) - 5-14 year: 7 (3) - 15-49 year: 25 (9) - 50-64 year: 16 (6) - ≥ 65 years: 7 (3) - Unknown: 8 (3) - P value < 0.001	- Female Positive/Negative: 129 (17)/628 (83) - Male Positive/Negative: 138 (20)/555 (80) - P value 0.159
A. Annamalay (2017)	- RV in autumn and lowest in winter (42.7% vs. 13.4%) - RV-A spring (43.3%) and autumn (31.7%)/ summer (15.0%) and winter (10.0%). - RV-C autumn (49.4%) and spring (11.2%).	- One respiratory virus (92%) - Human Rhinovirus (RV): (53%) - RV-A: 60 (38.2%) - RV-B: 8 (5.1%) - RV-C: 89 (56.7%)		Age in months: median (SD) - RV-A (n=60): 16.5 (18.6) - RV-C (n=89): 24.0 (15.0) - P value 0.737 Age group <12 months: n (%) - RV-A (n=60): 22 (36.7%) - RV-C (n=89): 24 (27.0%) - P value 0.209 - RV-C accounted for a higher proportion of RVs in the 2 to <3 age group than in the 4 to <6 age group (82.1% vs. 17.9%; P= 0.021).	Gender Male -RV-A (n=60): 37 (61.7%) -RV-C (n=89): 59 (66.3%) -P value: 0.563
S. Marcil *et al* (2018)	- A peak in winter: 62% (76/122). - Spring, 15% (18/122) of viral infections, - Summer 15% (18/122) - Autumn 8% (10/122). - The infections with RSV were diagnosed between mid-January and at the end of April with a peak in February: 13 cases detected in winter and one case in spring (p= 0.008). - The human metapneumovirus circulated exclusively in winter (p=0.002).	- One viral respiratory pathogen 95 of 147 (65%) - Multiple infection 18 (12%) - 41 samples (37 with two pathogens and 4 with three pathogens) - The overall positivity rate (PR) for any respiratory virus was 72% in children and 45% in adults. - 34 (28%) cases of rhinovirus infections - 21 (17%) cases of influenza infections - 16 cases of influenza A(H1N1) - 2 cases of non-H1N1 influenza A - 3 cases of influenza B - 15 (12%) cases of RSV A/B - 14 (11%) cases of human metapneumovirus A/B, - 11 (9%) cases of parainfluenza virus including eight parainfluenza 3, two parainfluenza 4, one parainfluenza 1, - Nine (7%) cases of enterovirus - Seven (6%) cases of adenovirus - Seven (6%) cases of coronavirus including two 229E coronaviruses, two HKU coronaviruses, two OC43 coronaviruses and one NL63 coronavirus - Four (4%) cases of bocavirus		- Children exhibited a significantly higher positivity rate compared to adults (p = 0.002). - Significant differences were observed in the presence of viruses across age groups, particularly concerning RSV and parainfluenza. - RSV was detected in 14 cases (93%) among pediatric patients and only one case (7%) among adults (p=0.04). - These 14 RSV cases were exclusively within the age range of 0-2 years. - Additionally, 11 cases of parainfluenza virus were isolated solely in pediatric patients (p=0.034), distributed across various age groups. - Multiple viral infections were exclusively diagnosed among pediatric patients, particularly within the age range of 0-2 years.	-
N. Daoudi *et al* (2019)	-	- HRV: 89 (26.33 %) - ADV: 3 (0.88 %) - HEV: 1 (0.29 %) - Influenza virus (A/B): 3 (0.88 %) - Parainfluenza (1-4): 9 (2.66 %) - HMP: 11 (3.25 %) - Coronavirus: (43/229E/NL 63 only) 7 (2.07 %) - RSV: 29 (8.57 %)		-	-
K. Hattoufi *et al* (2020)	-	-Acute respiratory tract infections: Viral origin (95%). -Respiratory syncytial viruses (A and B): (49%) -Rhinovirus: (21%) -Coronaviruses 229E: (11%) -Human metapneumovirus (5%) -Influenza A: (3%) -Influenza H1N1: (1%) -Adenovirus: (2%) -Parainfluenza virus type: 4 (2%)		Positives cases n (%)/ Negatives Cases n (%)/p value Age in hospitalization -10–30 days: 50 (83) 10 (17) ->30 days–<4 months: 21 (80)/5 (20) --P value 0.4	Positives cases n (%)/ Negatives Cases n (%)/p value -Male: 44 (95.7)/2 (4.3) -Female: 27 (67.5)/13 (32.5) -P value 0.001
H. Ezzine *et al* (2020)	-	-The prevalence of SARI rose from one season to the next, increasing from 51.8% (n=704) to 64.8% (n=819). -There was a significant increase in the overall positivity rate during the 2017/2018 season compared to the 2016/2017 season, with rates of 20.1% and 10.4% respectively (p<10^-6^).		SARI Positive n (%)/ILI Positive n (%) OR IC 95% P value - Age > 65 years - 30 (23.3%)/99 (76.7%) Age > 65 years -7 (2.8%)/245 (97.2%) -10.6 [4.5-24.9], <10-11	SARI Positive n (%)/ ILI Positive n (%) OR IC 95% P value Male sex -Yes 79 (61.2%) -No 50 (38.8%)/ Male sex -Yes 105 (41.7%) -No 147 (58.3%) 2.2 [1.4-3.4], <0.001
N. Ighid *et al* (2020)	- RSV infection showed the most pronounced seasonality trends. - Indeed, it typically exhibits a clear seasonality pattern with obvious peak in January and lowest activity in September and November. - Concerning AdV and RV viruses, peaks were in December and November respectively, while the low number of cases infection by the other respiratory pathogens unable detection of any seasonality.	-61.9% were positive for at least one virus. -Respiratory syncytial virus (RSV): 53.8% -Rhinovirus (RV) and coronavirus (CoV): 5.8% -Nucleic acids of Para Influenza (PIV), Streptococcus pneumoniae (S.P.) and Staphylococcus aureus (S.A.): 3.8% -Adenovirus (AdV) and Influenza A (InfluA): 1.9%		-The most infected patients were under two years old. -Respiratory syncytial virus (RSV) 50% of positive cases: 3.8% (3 to 5 years) and null (6 to 15 years) -The other pathogens were distributed mainly between the two groups (0-2) and (3-5) years. -PIV was the only virus detected in the group of 6 to 15 years (1.9%).	-
A. Lamrani Hanchi *et al* (2021)	-	- One respiratory pathogen: 387 (72.5%) - More than one pathogen: 23.3%	- HRV:(n=201) - RSV: (n=124) - PIV: (n=35) - Influenza A: (n= 29) - Human metapneumovirus: (n=27)	-	The rate of positivity boys vs girls (n= 225/310, 72.6%; n=162/224, 72.3%; p= 0.947).
A. Lamrani Hanchi *et al* (2022)	-	Before COVID-19 N (%) -Human Rhinovirus: 211 (36) -Respiratory Syncytial Virus: 149 (25.4) -Parainfluenza: 36 (6.1) -Influenza A: 35 (6) -Coronavirus: 23 (3.9) -Human Metapneumovirus: 27(4.6) -Adenovirus: 23 (3.9) -Influenza B: 6 (1)	During COVID-19 N (%) -Human Rhinovirus: 127 (40.2) (p>0.05) -Respiratory Syncytial Virus: 65 (20.6) (p>0.05) -Parainfluenza: 27 (8.5) (p>0.05) -Influenza A: 5 (1.6) (p=0.002) -Coronavirus: 15 (4.7) (p>0.05) Human -Metapneumovirus: 6 (1.9) (p=0.039) -Adenovirus: 10 (3.2) (p>0.05) -Influenza B: 0 (0) (p>0.05)	-	-
Z. Regragui *et al* (2022)	Positives -First Quarter: 90 (80.35) -Second Quarter: 9 (8.03) -Third Quarter: 0 (0) -Fourth Quarter: 13(11.60)	Positives: 11.89% (112/942) (Children under 5 years old)	All influenza confirmed cases, - Influenza AH1N1pdm09 68.75% (77/112), - AH3N2: 15.17% (17/112), - B: 16.04% (18/112)/ 94.44% (17/18): Influenza B/yamagata	The detection rate for influenza: -6 and 23 months (47.32%; 53/112) -2 and 5 years (30.35%; 34/112) -Under 6 months (22.32%; 25/112).	Positives -Male: 71 (63.39) -Female: 41 (36.60)
A. Bimouhen (2022)	- Viral circulation extends throughout the year. - A concomitant circulation of influenza and RSV viruses from November to April, with peaks during the months of December-March. - HAdVs and HRV circulated throughout the year with peaks during the winter months.	-	Detection rate: 70.8% - Influenza: 612 (30.5%) - RSV: (n = 359, 17.9%) - HRV: (n = 263, 13.1%) - HAdV: (n = 124, 6.2%) - PIVs: (n = 107, 5.3%) - HCoV (n = 94, 4.7%) - Human bocaviruses: (HBoVs) (n = 92, 4.6%) - HMPV: (n = 74, 3.7%)	SARI <5 years, N = 537 -Inf A/B: 53 (9.8) -RSV: 228 (42.4) -HAdV: 70 (13.0) -HRV: 81 (15.1) >5 years, N = 270 -Inf A/B: 71 (26.3) -RSV: 10 (3.7) -HAdV: 8 (2.9) -HRV 36: (13.3)	-
Z. Regragui *et al* (2023)	-	The prevalence of respiratory pathogens: 43% (n=72) -Human Rhinovirus: (HRV) (38%) -Human Coronavirus: OC43 (37%) -Human Parainfluenza3: (HPIV3) (12%)		-	-

## Discussion

The present study aimed to identify all the viruses circulating in Morocco, through a systematic literature review. Different seasonal flu serotypes circulate in Morocco each year, including B, H1N1, and H3N2. In this sense, a study carried out in France identified Forty-one (41/107; 38.3%) had influenza: 38 (92%) were positive for influenza A ( A(H3N2)) and three (8%) for influenza B/Victoria (Masse *et al.*, 2017) .

A(H1N1) pdm09 was dominant and exceeded 30% in almost all the studies in this review, especially for the studies carried out from 2009 in Morocco. This is in agreement with a study that aimed to track influenza activity in Bangkok, Thailand, between June 2009 and July 2012. Results identified that influenza virus infection A pH1N1 was detected in 42% (n=2697) samples, and were the predominant strain between 2009 and 2010 (Prachayangprecha *et al.*, 2013). On the other hand, another study carried out in Singapore which aimed to monitor the prevalence of influenza in 2009 revealed that 24.0% had the H3N2 subtype, 1.6% had the seasonal H1N1 subtype and 2.7% had influenza B (Leo *et al.*, 2010), and a slight prevalence in India between 2009 and 2010 *(Mukherjee et al.*, 2010).

Regarding RSV, the most common cause of lower respiratory tract infection in infants and children worldwide, eight studies in this review looked at the prevalence of RSV and the results revealed a prevalence ranging from 18% to 36.47% in the pediatric population, similar findings were identified in infants and children worldwide in Spain (García-García *et al.*, 2006), and in another of three Southeast Asian countries (Wertheim *et al.*, 2015). Another study carried out in Sri Lanka aimed to describe the prevalence and characterization of RSV, the results revealed that RSV was the most common virus detected in children in Sri Lanka between 2016 and 2018, co-infections with RSV-A and RSV-B subtypes were noted during circulation of this virus, which was not identified in the results of our review. The same study also confirmed that RSV activity occurred throughout the year in the study area over the same period (Divarathna *et al.*, 2022).

Our review, while focusing on individual viral pathogens, acknowledges the potential role of co-infections in disease severity and outcomes. Limited data exists within the included studies regarding the prevalence and impact of co-infections in Morocco. However, studies from other regions, such as the one in Vietnam, highlighted that co-infections with AdV can occur in a significant proportion of cases. Further research is needed to understand the specific patterns and clinical implications of viral co-infections in the Moroccan context (Pham *et al.*, 2020).

In the present review, it was observed that RSV exhibited a pronounced seasonal pattern, with a high prevalence during the winter season. Cold temperature and increased precipitation rates facilitate the proliferation of viruses. A comparable pattern of RSV prevalence has been documented in the eastern Indian state of Odisha, where RSV infections exhibit seasonal fluctuations, peaking during the rainy season and continuing into the winter season (Panda *et al.*, 2017).

Compared to age distribution, the results of the present review revealed a high prevalence of RSV in male children younger than 12 months and in men. Comparable variations in age and gender vulnerability to RSV infection have been previously documented in various other geographic areas (Sangaré *et al.*, 2006; Oliveira *et al.*, 2008; Zhang *et al.*, 2010). Risk factors for RSV infection include young age, premature birth, cold exposure, smoking, underlying medical conditions and male gender (Halasa *et al.*, 2015).

Our review reported a prevalence of RV in children that exceeds 50%, similar findings identified in a recent study in France performed between May 1, 2011 and April 30, 2016 which found RV accounts for 34.3% of circulating viruses in children during this period (Visseaux *et al.*, 2017). Another study in Peru showed a prevalence of 41.86% cases that were diagnosed with RV, of which 73.07% were RVC, 16.67% were RVB and 10.26% were RVA. Infections caused by RV were more common in male patients (57.26%) and in the 0-5 month group (48.72%) (Castañeda-Riveiro *et al.*, 2022). in three studies carried out on VR in Africa. The results showed varying prevalences of RV-A compared to RV-C; one reporting equal prevalence (Onyango *et al.*, 2012), one with RV-A as the most common (Esposito *et al.*, 2012) and one with RV-C predominating (Smuts *et al.*, 2011). However, it should be emphasized that the populations of the three studies presented differences regarding their age and clinical manifestations.

Distinct seasonal patterns were evident for RV-A and RV-C, with RV-A reaching its peak during the spring months and RV-C exhibiting its peak in the autumn season. Findings from various global studies have corroborated the observation that RV-C is most commonly encountered during the autumn season (Lau *et al.*, 2007; Miller *et al.*, 2009; Richter *et al.*, 2015), and RV-A is frequently found to be most prevalent during the spring season (Miller *et al.*, 2009).

Another study aimed to compare the two viruses RSV and RV showed that RV was the second causative agent isolated in 14.7% of children hospitalized with wheezing in Suzhou, China, after RSV (21 .0%) (Sun *et al.*, 2016) . Another observational study, conducted over a period of one year (June 2021-May 2022) revealed that the most commonly isolated virus was human RV (55.8%) followed by RSV (23 .5%) (Agarwal *et al.*, 2023).

A low prevalence of AdV was mentioned in two studies in the present review (2% and 17%). In parallel, a study carried out in Lebanon revealed an AdV prevalence of 25.32%, the infected patients were mainly aged 24 to 35 months or 4 to 11 months (Zaraket *et al.*, 2020). Another recent study published in 2021 among the population in Vietnam showed the detection of AdV in 12.96% of children hospitalized for community-acquired pneumonia , same study found that 36.1% of cases with AdV were co-infection with other respiratory viral pathogens *(Pham et al.*, 2021).

Socio-economic factors can profoundly impact the transmission and severity of respiratory viral infections. Overcrowding, inadequate sanitation, and limited access to healthcare may increase the risk of infection and contribute to poorer outcomes. While our review did not directly address these factors, it is plausible that they play a significant role in the epidemiology of respiratory viruses in Morocco, particularly in vulnerable populations. Future research should explore the correlation between socio-economic status and the prevalence and severity of respiratory viral infections.

This review found that the proportion of HPMV in children varies between 5% and 9.1%. A review of studies published between August 2017 and August 2019 concluded that the overall prevalence of HMPV in children ranges from 1% to 86% depending on the country (Van Den Bergh *et al.*, 2022). Another study showed that the economic cost of this type of virus is estimated in the United States at $277 million each year (Davis *et al.*, 2016).

The studies in this review, carried out during the covid-19 pandemic in Morocco, revealed a significant change in the epidemiology of viral respiratory pathogens. A significantly lower positivity rate during the COVID-19 period was found (p=0.006), especially in infants under 6 months (p=0.008). There was a substantial absence of detection of RSV and influenza A during the winter season following the outbreak of the pandemic (p < 0.05; p = 0.002 respectively) (Hanchi *et al.*, 2022) , same results mentioned by several studies of the literature, that revealed a significant decrease in the hospital admission due to non-COVID-19 respiratory infection, particularly those due to RSV and influenza, in France, Asia and Latin America (Fourgeaud *et al.*, 2021; Friedrich *et al.*, 2021; Rodgers *et al.*, 2021; Yum *et al.*, 2021). These changes could be explained by the impact of the implementation of preventive measures related to the COVID-19 pandemic on the transmission of respiratory pathogens (Chan *et al.*, 2020; Oster *et al.*, 2021).

Finally, and compared to the parainfluenza virus, the proportion of parainfluenza virus has been estimated between 2% and 10.29% in the studies of this review which are carried out in children. A study which aimed to determine the distribution of ARI in children admitted to a general hospital in Sri Lanka identified that parainfluenza virus (PIV) was detected in 40/340 (11.76%) (Rafeek *et al.*, 2018).

Based on the findings of this review, we recommend that public health stakeholders in Morocco prioritize several key actions. These include implementing enhanced surveillance systems to monitor the circulation of respiratory viruses, including the identification of emerging strains and co-infection patterns; prioritizing vaccination efforts for influenza and considering targeted RSV prevention strategies for high-risk infants; promoting hand hygiene and respiratory etiquette through public health campaigns, particularly during peak seasons; and investing in further research to investigate the impact of socio-economic factors, co-infections, and specific risk factors on respiratory viral infections in Morocco. These measures are crucial for mitigating the burden of respiratory viral infections and improving public health outcomes in Morocco

## Conclusions

This review suggests that Pandemic and Seasonal Influenza, RSV and RV have a considerable prevalence in the samples studied in the different cities of the Kingdom of Morocco, although the data on the circulating viral respiratory pathogens are limited, because several selected studies have focused more on infections in children and hospitalized patients. Further studies and surveillance incorporating laboratory molecular typing in adults and non-hospitalized patients are needed to determine the overall burden of infection of the different viruses in our country.

### Conflict of Interest Statement

The authors declare that there are no conflicts of interest regarding the publication of this article. The research was conducted without any financial or personal relationships that could be perceived as influencing the work.

### Author’s contribution

Kharbach Ahmed and Baba Mohamed Amine contributed equally as co-first authors for this article.

**Abbreviation list:**
ILI: Influenza-Like Illness;
SARI: Severe Acute Respiratory Illness;
RT-PCR: Reverse Transcription Polymerase Chain Reaction;
BAL: Bronchoalveolar Lavage;
HMPV: Human Metapneumovirus;
RSV: Respiratory Syncytial Virus;
ADV: Adenovirus;
HRV: Human Rhinovirus;
CoV: Coronavirus;
BoV: Bocavirus
PICOS: Population, Intervention, Comparison, Outcome, Study design;
PRISMA: Preferred Reporting Items for Systematic Reviews and Meta-Analyses;
JBI: Joanna Briggs Institute;
qRT-PCR: Quantitative Reverse Transcription Polymerase Chain Reaction;
FA-RP: Film Array Respiratory Panel;
CSP: Clinically Severe Pneumonia
